# Medical and Dental Examination of School Children

**Published:** 1908-12-15

**Authors:** Thomas B. Cooley

**Affiliations:** Detroit, Mich.


### MEDICAL AND DENTAL EXAMINATION OF SCHOOL CHILDREN.

BY THOMAS B. COOLEY, M.D., DETROIT, MICH.

Address to Michigan First District Dental Society, Nov. 12th, 1908.

This is the first time that I have had the pleasure of meeting with you, and I want to say that I consider it a distinguishing privilege to speak on this subject before your Society which, so far as I know, has taken the most advanced ground that has yet been taken in this country regarding dental examination of school children. I have not prepared a written paper and do not mean to talk long. I shall endeavor to present a little of the history of school inspection showing what has been done in other places, and what we are trying to do and hope to do later on in Detroit.

There seems to be an impression abroad, since we have made a good deal of stir about medical school inspection in this country, that we are the pioneers or leaders in this matter in America. This is not true. There has been some medical school inspection in some places in Europe since before the middle of the last century. There has been regular methodical school inspection in Belgium since 1874, and in France since 1884, and in a good many other countries since previous to 1890. Medical inspection in this country practically began in Boston in 1894.

The movement toward medical school inspection comes from two sources. The first and most obvious source is from the side of public health authorities, because since we began giving attention to public sanitation we have realized that the schools were the great centers for the spread of contagion. This results from the fact that the contagious diseases are more common amongst children, and from the way they are associated in our public schools, they naturally become the great centres for spreading diseases. Our local or state governments, in view of the fact that they insist that the children shall be in school, are therefore responsible for the children while they are in the school, and it is their duty to see that the school is a reasonably safe place for the children. This was fully recognized years ago when the schools were frequently closed on account of contagious epidemics. Every community is also responsible for the spread of contagion in its midst.

In most places, in this country at least, the medical inspection is carried along on this line only. But there is another side, the educational side, or the making the most that can be made out of the school children intellectually. Everything that may have a bearing upon the welfare of children in school is of importance. Few things are of more importance on that side than the physical ability of the child to make the best of the instruction given him. It is very obvious that the child who is deaf or blind cannot get all that others will out of school. These are extreme cases but the minor inefficiencies in vision and hearing give perhaps the next most obvious to interfere with the child's progress in school. There are many other things less obvious than these, and yet almost equally important,—the child who does not breath well; does not get a decent amount of oxygen into its lungs is consequently more or less poisoned, dull and stupid and seldom attends well to his school work, consequently he does not get along in school as he should. The child that is not well fed, whose nutrition is not up to normal, cannot be expected to do the brain work that he ought to do, and, as the pressure of school work increases, as it does from year to year, the disability depending on some physical defects becomes very marked. There has been recently, a very decided sanitary advance made through the medical inspection of school children for physical conditions and physical defects.

I have said that the history of school inspection in this country is quite recent compared with what it is abroad; in fact, we have not gone so far as they have abroad. In 1898, four years after we first had any school inspection here, Japan had a law providing for a salaried school inspector for every public school in the country. A great many countries are ahead of us so far as any general organization of medical inspection of schools is concerned; Russia, England, even some South American Countries I believe are ahead of us so far as any general provision for this medical inspection is concerned.

In this country it started, as in fact it did practically everywhere, with the exception of Japan, by a voluntary movement. In Boston there were a few voluntary inspectors at first; later they were apointed on a salary, and gradually their system has been built up just as it has later here in Detroit. The legislature of Massachusets now has a law such as Japan has, providing for the medical school inspector for every public school.

The best organized system in this country is in New York City, although some remarkable work has been done in Cleveland. I think the work done in Cleveland stands next to what has been done in New York. Connecticut has a law permitting municipalities to appoint school inspectors and inforce an inspection. New Jersey also has such a law. Vermont has a law, passed in 1904, providing for compulsory eye, ear, nose and throat examination of children. These are the only state laws in the country on this subject. Everywhere else outside of these States it has been undertaken and encouraged by the public health authorities; they simply have assumed the right to do it and have gone ahead. There are, altogether, 20 cities in the United States outside of Massachusetts, that have a school inspection. The amount of inspection varies greatly in the different cities, from next to none to very complete systems.

In New York, as I have said, the system is probably the best we have any where in the country. There are, under the supervision of the Board of Health and the Chief Examining Inspector of schools, 166 salaried inspectors, who receive $1,200 a year each. These 166 inspectors each have on an average 3,151 children under inspection. The total salaries of the inspectors alone in New York amounts to $199,200; this is exclusive of the salary of the Chief Inspector, which I believe, is $2,500, and also exclusive of the salaries of the 75 nurses, each of whom gets $900 a year. The Board of Health maintains free clinics for the treatment of skin diseases of school children, so that the expenses in New York City must be at least $300,000 a year for school inspection and care of their school children's teeth. As compared with this, I might say that here in Detroit, we have 27 inspectors, taking care of an average of 1398 children apiece, and their total salaries amount to $6,750, and there is no other expense here except for two nurses—who are paid $75.00 per month for ten months, or $750 a year. This would give a total outlay of $8,250, as compared with some $300,000 in New York. The cost per capita in New York is 38 cents for each school child; in Detroit it is 17.8 cents Now in New York they have not only an inspection of contagious diseases, but a regular and complete physical examination of each child, at least once a year to ascertain the state of the nutrition in general, especially diseases of the nose, throat and the glands, for diseases of the heart, lungs, deformities of any kind, diseases of the skin, and such special diseases as chorea, which seems to be of importance in New York. There probably is nowhere a more complete system of medical inspection and their statistics have shown it to be of very great value.

In Detroit, we started just as they did in Boston, with a system of voluntary inspection for a time and then with quite a few inspectors on salary of $25.00 a month. At first all of the news-papers were down on the idea, our health officer showed me to-day an article published in one of our papers at the time he first asked for the salaries of seven school inspectors which he had then at $25.00 a month. The article was an inconceivably brutal attack upon the health officer. They called him a grafter, political thief, and all sorts of things for asking for pay for those seven school inspectors. The system of salaried inspectors is now in its fourth year, and all the news-papers are now heartily in favor of the system and not only that but the teachers in the schools and the public in general have shown a great interest in the work. There was, at first, a good deal of opposition on the part of the parents, to this inspection of their children in the schools; at first they were very unwilling to have it done, but they have discovered that it pays. It has now come to pass that if the medical inspector happens to be sick, or called off for a day, the parents of the child, as soon as the child comes home and they find the doctor was not there, begin to send in complaints to the health office that the school doctor did not show up. Not long ago there was a howl in the paper from some man because his child was in a school where the child developed scarlet fever; he did not complain of being sick in the morning and was not sent to the inspector. At noon, however, he was taken sick, and it proved to be scarlet fever—so that, on the whole, the attitude of the press and public has changed very decidedly.

It has been rather difficult to get money here in Detroit to carry out the work as we would like. I am not speaking officially for the Board of Health, but I have been interested in the work for some time, and so am speaking for all the inspectors as well as in a way for the health officer. We would like to do a good deal more than we have been doing, but it has been so difficult to get the money that we have gone rather slowly, because we felt that it was best to have the public satisfied that its money was well spent.

Up to this year the work nominally has been purely for the exclusion of contagious diseases. To be sure, some of the inspectors have, right along, sent home notes to their parents in cases where children had obvious physical defects, advising that something be done for them, and have been able to get something done in certain cases, but this has not really been official, and our system did not provide for it. This year some attempt is being made, not only to give a routine physical examination of the children, but to have tickets sent to us by the teachers of children who have apparently some physical defects, for more careful examinations, and we have a separate card to send home for those things. We have had before simply the exclusion card, which excluded a child for contagious diseases; if we wanted to recommend something else, we had to scratch out that and write something else on the card. We make use of both cards now, and also have a separate system of report blanks for the two.

The average inspector has four schools—some have five. We cover not only the public schools but the parochial schools (for quite a while we did not, but we finally managed to stir up the Bishop and get these also included). The inspector goes to each school in the morning; all the children who have been absent on account of sickness; all who are complaining of feeling ill in any way, and all children that the teacher thinks show any signs of illness are sent to the inspector. He examines all such children—and also any children that the teacher thinks may have physical defects for correction. The inspector after examining all such children will exclude all who have contagious diseases of a nature which makes it unsafe for them to be at school. There are certain forms of skin disease which are mildly infecticus; only so when they are not properly looked after, where, if the circumstances are favorable, the child may be allowed to stay in the school, but if the parents obstinately refuse to take proper care of the disease; if the child, for instance, has a sort of skin eruption which is easily spread by scratching and getting the infection on his fingers, etc., and he will not let it alone, we exclude him from school. If, on the other hand, when his attention has been called to the trouble and is told how to take care of it, if it is taken care of, he can stay in school. A certain number of cases, of course, are excluded always—exophthalmatas, scarlet fever, chicken pox, measles, etc. Tonsillitis is also excluded. Then there are the physical defects that we find. If they need correction, we send home this recommendation card to the parents, and of course this system is still new here, but we hope to be able to follow these cases and find out how much has been done for them. That is, in the schools where we have no nurses.

The nurses are a very great aid to school inspection, and one of the things we especially hope for is more nurses. The nurses are being used now especially in the schools mainly attended by the poor and ignorant classes. They treat certain forms of skin diseases among the poor people, who cannot afford to go to a doctor, right in the school. The treatment is simple and can be perfectly carried out. In other cases, where the treatment should be at home or at the hospital, and the parents are not doing anything for them, the nurse follows such children to the home. She explains the thing to the parents; if it can be taken care of at home, she shows them how to do it and sees that it is done. If the child needs to be taken to the hospital and the parents are too busy to take the child to the hospital, the nurse takes it, and if the child needs an operation, the nurse will stay with the child until the operation is over and the child is fit to go home. Sometimes the nurse will get several operations performed in one week, where the parents could not possibly manage it. Really in some schools the nurse's services are worth more on the whole, than the medical inspector's. That would not be true every where, but es. pecially where skin diseases are prevalent the nurse can do no more good than any one else. Of course the nurse cannot diagnose the dangerous contagious diseases and exclude them from the school, but for the practical treatments they really are of more use than any body else, and we much need more of them.

Now this is in general about what our system is at present. It might be of some interest to know just how much was done last year; for instance in the line of excluding contagious diseases in the schools. This is the report from July, 1907, to July, 1908:

Total number examined in schools, 46,713. Total number excluded, 2,756. Of this were: Scarlet Fever, 5, which is rather low; the average would be more than that of cases picked up in the schools. I have so far this year in the schools I examined found two cases of scarlet fever. Diphtheria was only 4 last year. In an average year it would be many more Tonsillitis, 441. Measles, 65. German Measles, 9. Mumps, 567.; we had a very extensive outbreak of mumps last year. Small-pox, 2. Chicken-pox, 93. Whooping cough, 65. Ring-worm, 46. Scabies (itch), 39. And other unclassified causes for inspection, 452.

That is probably, on the whole, a pretty fair percentage. That is the number of exclusions under a system like ours. Twenty-seven and 56 is a fair percentage of all the children examined. I think it depends a good deal upon the class of children as to what percentage you will have to examine and exclude.

There has been a good deal of controversy as to whether it was wise to leave the selection of the children to be examined to the teachers, or whether the inspector should also go to the rooms of the school and glance over the children and pick out cases. I am inclined to think that the system we have here is the only one that can be worked out here as a routine system.

I said at the start that I believed this Society had made a more advanced start than any society in this country on the question of dental inspection of school children. There are only a very few meager statistics regarding the dental inspection of school children, except in certain European countries. The most thorough and the best systems are in Germany where there are free dental clinics for care of the teeth of the school children. Their statistics show that somewhat over 90 percent. of all the children examined in these schools have defective teeth, as high as 97 percent. Statistics from Scotland show the same thing. In one town in Scotland where the routine examination of school children was made, they did not find a child whose teeth had not had or was in need of attention from the dentist, out of some 2,000 children. That, I think, is a very remarkable report.

In this country I do not find anything practical in the way of routine statistics. There have been some special examinations made. I should say that I have seen some recent partial reports from Canada, and they show a curious contradiction to those foreign statistics. For instance in both Montreal and Chicago the statistics showed that only 27 percent. of the children examined had bad teeth. It does not seem to me that it could have been a very complete examination of these children to show such a percentage. I do not believe that in any large city you can find a lot of school children with only 27 percent. having bad teeth. I think the statistics are unreliable and correspond with the reports of eye troubles in these places; the proportion of eye troubles reported was something less than 3 percent of all the children examined. Of the examinations that have been made here, of the eyes of school children, the percentage would run to 40 or more, where there was evident defective vision or some definite eye trouble, so that I feel very sure that all these statistics must be rather low. I imagine that they were made up by simply the ordinary routine school inspection rather than skilled specialists, and in that serious case many times have been overlooked.

There are three cities in Massachusetts which have a sort of dental inspection of the school children. The children's teeth are examined regularly, and they have report blanks somewhat similar to the one Dr. Giffen has planned to use in Detroit, to show the condition of the teeth. I know of no city in the country where a dental society has taken up this subject in the way you have, and where a dental clinic has been started especially to take care of these cases found in the schools.

We want a complete physical examination of all the children in our schools, to include eyes, ears, nose, throat, teeth and everything else that is of any importance from the educational or sanitary standpoint, and we shall need more money to do it. We want more nurses, and we shall need money for that. We believe the best way to get money is to show the people what we can do with it. To that end I proposed to Dr. Kieffer some time ago that I take one of the school nurses and make a complete inspection of a small lot of school children in a selected school, to find out just exactly what proportion of them had physical defects, and what they were, and then what we could do to get these defects corrected with the co-operation of the nurse. When we found that a Committee from this Society was also moving in that direction and for the establishment of a dental clinic, we, of course, immediately welcomed this chance of having our examination more complete. We hope to start this work with the co-operation of your clinic for the teeth, and of the various free medical clinics around town for the children who cannot afford to pay. It will be necessary to exclude in time that proportion of children who can afford to pay from the free clinics. With the co-operation of these free clinics for those unable to pay, and with the co-operation we hope to get from the dentists and physicians, and from those who are able to pay, we shall get enough of these physical defects of one kind and another corrected, so that after some months we shall have cases enough to enable us to correct our statistics by tabulating our cases, and to show that we can do that which the men who provide the funds will realize the necessity of providing more funds, and then we shall be able to have the careful and thorough examinations made obligatory in all schools and for every child.

I might say that I do not feel at all certain, so far as the dental side goes, that this is going to be necessary always, or advisable perhaps always to have a routine inspection of the school children by dentists. That is to say, of course I realize it would be better so far as the thoroughness of the examination goes, but it is some question in my mind, whether having two sets of examiners in the schools would not make it so cumbersome as to bring it into disfavor with the school authorities. I am not all certain that after we have worked it out and have gone through a sample lot for the collection of careful statistics, we won't find it possible to get what is necessary done simply in the routine medical examination by the school inspectors stating whether or not the oral hygiene is good or bad, the teeth are decayed or not decayed—whether, in other words, the detailed examination of the teeth by the dentist will be necessary when it comes to the long routine examination. That is still to be worked out, and I think it very desirable that we should get this very thorough routine examination of a small lot, in the first place, to show just what the percentage is and what we can do for them. The other things will work themselves out later. I think it is very desirable that we continue this dental inspection, and I think the dental clinic will do a great deal of good.

It is not always so obvious to the layman, teacher or parents, just what bearing the state of the child's teeth has on his progress in the school, but I think it is pretty obvious to all of you, as it is to me, that anything serious troubling the teeth is bound, in the end, to affect the child's nutrition, and certainly anything affecting the child's nutrition will affect his progress in school.

I think really it is going to be one of the most important things in the physical inspection of the children, coming next to the eye, ear, nose and throat.

Another thing I want to say is that I have had the question raised once or twice by very intelligent people who talked to me about it as to the economic question as to whether, granting all the benefit you can get from this sort of medical inspection of school children, whether the public ought to pay for it or not. It does seem to me that there is but one side to such a question. It may sound well to say that the parents ought to pay for keeping the children in condition, or that the child ought to go to the public commission, or this, that and the other thing, but after all, there is no question that the better physical condition the child is in his school years, the more he is going to get out of the school, and the better citizen he is going to make, and the more of a producer of wealth in the end, and if we think it worth while to teach the children anything at all, we want to have them get all they can out of it, because we are going to get more back out of them later on. There is no question in my mind but that the public ought to pay all that is necessary to have and to keep our school children in the best possible physical condition.

